# Physiological control of water exchange in anurans

**DOI:** 10.1002/ece3.8597

**Published:** 2022-02-10

**Authors:** Lee A. Lemenager, Christopher R. Tracy, Keith A. Christian, C. Richard Tracy

**Affiliations:** ^1^ Department of Biology University of Nevada Reno Nevada USA; ^2^ 149877 Organic Program Washington State Department of Agriculture Olympia Washington USA; ^3^ Department of Biology California State University Fullerton Fullerton California USA; ^4^ Research Institute for the Environment and Livelihoods Charles Darwin University Darwin Northern Territory Australia; ^5^ 8790 Philip L. Boyd Deep Canyon Desert Research Center University of California Riverside Indian Wells California USA

**Keywords:** aquaporins, frogs, hydroregulation, water exchange, water potential

## Abstract

Research on water exchange in frogs has historically assumed that blood osmotic potential drives water exchange between a frog and its environment, but here we show that the “seat patch” (the primary site of water exchange in many anurans), or other sites of cutaneous water uptake, act as an anatomic “compartment” with a water potential controlled separately from water potential of the blood, and the water potential of that compartment can be the driver of water exchange between the animal and its environment. We studied six frog species (*Xenopus laevis*, *Rana pipiens*, *R*. *catesbeiana*, *Bufo boreas*, *Pseudacris cadaverina*, and *P*. *regilla*) differing in ecological relationships to environmental water. We inferred the water potentials of seat patches from water exchanges by frogs in sucrose solutions ranging in water potential from 0 to 1000‐kPa. Terrestrial and arboreal species had seat patch water potentials that were more negative than the water potentials of more aquatic species, and their seat patch water potentials were similar to the water potential of their blood, but the water potentials of venters of the more aquatic species were different from (and less negative than) the water potentials of their blood. These findings indicate that there are physiological mechanisms among frog species that can be used to control water potential at the sites of cutaneous water uptake, and that some frogs may be able to adjust the hydric conductance of their skin when they are absorbing water from very dilute solutions. Largely unexplored mechanisms involving aquaporins are likely responsible for adjustments in hydric conductance, which in turn, allow control of water potential at sites of cutaneous water uptake among species differing in ecological habit and the observed disequilibrium between sites of cutaneous water uptake and blood water potential in more aquatic species.

## Graphical abstract

Research on water exchange in frogs has historically assumed that blood osmotic potential drives water exchange between a frog and its environment, but here we show that the “seat patch” acts as an anatomic “compartment” with a water potential controlled separately from water potential of the blood, and the water potential of that compartment can be the driver of water exchange between the animal and its environment. Different controls of the seat patch water potential explain adaptations to different ecological habitats.

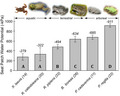

## 1 INTRODUCTION

Amphibians can be found in habitats ranging from fully aquatic, to fully terrestrial or arboreal (Hillman et al., 2009). Some habitats are xeric and can potentially cause rapid dehydration, and death due to desiccation. However, some amphibians employ physiological and behavioral mechanisms both to prevent water loss and to increase water uptake (Bentley, 1971). Understanding the successes of amphibians in environments differing in degree of terrestriality requires understanding the intricacies of adaptations for water exchange in different hydric environments (Tracy et al., 2014).

Nearly all anurans (frogs and toads) have a very thin stratum corneum relative to other terrestrial vertebrates (Drewes et al., 1977; Shoemaker & McClanahan, 1975) and must maintain hydrated skin to survive (Duellman & Trueb, 1994; Jorgensen, 1997a). Water loss rates from amphibians are high relative to other terrestrial vertebrates, and can be lethally rapid (Hillman et al., 2009; Jorgensen, 1991, 1997a, 1997b; Young et al., 2005). Rather than drinking, anurans replace water losses by efficiently absorbing water through their skin (Hillman et al., 2009; Jorgensen, 1994; McClanahan & Baldwin, 1969; Sinsch, 1991; Tracy et al., 2013).

Many frogs are able to absorb water rapidly through a highly vascularized area of ventral skin called the “seat patch”, which is associated with both behavioral and physiological adaptations for water uptake (Bentley & Main, 1972; McClanahan & Baldwin, 1969). The size and location of this site of facilitated water absorption varies among species of terrestrial frogs and toads (Ogushi, Tsuzuki, et al., 2010). Behavioral adaptations consist of body posturing, so that the seat patch is adpressed against a wet environmental surface from which water can be absorbed (Heatwole et al., 1969). Physiological adaptations of the seat patch include controlling rates of water uptake via antidiuretic hormones (Bentley, 1969; Cree, 1988; Hillman et al., 2009; Tracy & Rubink, 1978), which can influence the permeability of the seat patch largely by poorly understood mechanisms that include changes of skin conductance (Tracy, 1976, 1982), changes to blood flow (Viborg & Hillyard, 2005), and seat patch water potential (Hillman et al., 2009; Tracy & Rubink, 1978).

In addition to the need of anurans in terrestrial environments to absorb water from their desiccating environments, anurans in aquatic environments face the challenge of restricting osmotic absorption of water from their environments. If the driving force to water absorption is the osmotic gradient between blood osmotic potential and a freshwater environment, then physiological adaptations are required to prevent excess water uptake for both completely aquatic frogs and other anurans that are unavoidably in water for extended periods. Completely aquatic frogs (e.g., Pipidae) do not have a specialized seat patch (Ogushi, Tsuzuki, et al., 2010). Nevertheless, the frogs do take up water across their ventral skin (as evidenced below). For the sake of simple expression, we will use the term “seat patch” in reference to the ventral sites of physiological control of water absorption whether or not they are specialized regions with high capillary density.

We studied the seat patch water potentials of six species that differ in use of habitat. The African clawed frog, *Xenopus laevis*, is a fully aquatic species while the American bullfrog, *Rana catesbeiana*, is semi‐aquatic. The Northern leopard frog, *Rana pipiens*, and the Western toad, *Bufo boreas*, are terrestrial species. The Pacific chorus frog, *Pseudacris regilla*, which can be found both in trees and under logs, and the California tree frog, *Pseudacris cadaverina*, which is often found on rocks near streams, are considered arboreal species. We hypothesized that because the six frog species are found in a wide range of hydric environments, there would be advantages to them to be able to regulate their water uptake in ways that are adapted for their different hydric environments. Thus, highly terrestrial species would benefit from a seat patch water potential that is much lower than that of the environment to facilitate rapid water uptake or uptake from moist soils rather than free water (Tracy, 1976), but fully aquatic frogs would benefit from a seat patch water potential that is closer to that of water as a way to avoid excessive water uptake. Here, we report on experiments to measure seat patch water potentials of several frog species that differ in their ecological habit.

## 2 METHODS

### 2.1 Animals and lab conditions

Specimens (mean mass ± SD) of *X*. *laevis* (3.4 ± 1.0 g) and *R*. *pipiens* (52.1 ± 4.1 g) were purchased from commercial suppliers (*X*. *laevis* from Aquatic Plant Depot, Tampa, Florida, USA; and *R*. *pipiens* from Charles D. Sullivan Co., Inc. Nashville, Tennessee, USA). *Rana catesbeiana* (60.8 ± 12.8 g) and *B*. *boreas* (93.6 ± 14.7 g) were captured at Rancho San Rafael Park (39.550186°N, −119.828606°W; 4672 m) in Reno, Nevada, USA. *Pseudacris cadaverina* (4.2 ± 1.6 g) were collected from Orange County, California, USA, and *P*. *regilla* (5.4 ± 1.5 g) were collected from Limekiln Canyon Wash, Rinaldi Park, Northridge, Los Angeles Co., California, USA (34.27743°N; 118.56192°W; 329 m). Between experiments, all frogs were maintained in 38 L aquaria in a laboratory at the University of Nevada, Reno where air temperatures ranged from 21 to 26°C. The larger frogs were housed in groups of two to three individuals per aquarium, and the smaller frogs were maintained in groups of three to five individuals per aquarium.

### 2.2 Water exchange with the environment

The seat patch water potential was inferred from experiments using different environmental water potentials to find the conditions where frogs do not exchange water with the environment, that is, when the water potential of the environment and seat patch are equal (Tracy & Rubink, 1978). Prior to each experiment, frogs were placed in pure water for 2 h to allow them to become fully hydrated. Then, frogs were catheterized to remove any water in the bladder. The body mass of the frog with an empty bladder was then recorded as the “standard body mass” (McClanahan, 1972; Ruibal, 1962; Tracy, 1976), and then the frogs were dehydrated in a wind tunnel to 90% of their standard body mass which took from one to several hours depending on species and activity of the frogs. Once they reached this target level of dehydration, the frogs were placed individually into an apparatus similar to the one used in Tracy and Rubink (1978) consisting of a plastic container containing a sucrose solution so that only the ventral surface of the frog's body was in contact with the solution, and the rest of its body was exposed to the air. For the following hour, the frogs’ body masses were measured every 10 min after the frog was blotted dry with a paper towel. Each frog was tested in the various sucrose solutions, with at least 7 days between experiments. The order of testing began with pure water and progressed toward more negative water potentials.

Cutaneous evaporative water loss during the water uptake experiment was estimated from experiments using frog models made from 3% agar molded into the shape of the frog, which evaporates as a free water surface of the given size and shape (Spotila & Berman, 1976). Negative molds of each species of frogs were made by pouring dental alginate on live frogs that had been given MS222 (Tricaine methanesulfate) as an anesthetic to minimize the frog's movements while the mold was being made. Alginate sets in less than 1 min, so no harm is caused to the frog. Plaster of Paris was then poured into the alginate mold to create a positive mold of the frog. Latex was then painted onto the plaster of Paris mold in several layers to make a thick and durable negative mold that could be reused many times. A 3% agar solution was then poured into the latex mold and allowed to set. By this approach, the agar frog models were made to be the same size, shape, and posture as the living frogs from which molds were made. Water absorption by the resulting agar model was prevented by coating the venter of the model with fingernail polish approximately where the frog would be in contact with the sucrose solution. The agar frog model was placed in the experimental apparatus for measuring water uptake, but the liquid solution was not allowed to come into contact with agar frog model. Thus, the relative humidity in the apparatus was the same as that in experiments for measuring seat patch water potential. The body mass of the agar models was measured every 10 min.

The mean change in frog body mass in the apparatus reflects the sum of water uptake and evaporative water loss, so the water influx into the frog was obtained by adding the evaporative water loss (estimated from the agar models as described above) to the water exchange (measured in the apparatus described above). This calculated water influx was then plotted against water potential of the environment (sucrose solutions) to obtain the x‐intercept of this relationship, which is the point at which no liquid water was exchanged because the water potential of the seat patch was equal to the water potential of the sucrose solution. Data were discarded in cases when excessive frog activity resulted in questionable results. The bladders of the frogs were voided prior to being dehydrated to 90% standard body mass to decrease the likelihood of urination, but the data were discarded when either urination or defecation occurred during the experiments. Inspection of plots of water uptake as a function of sucrose water potential showed two patterns for each species except *P*. *cadaverina*: a sloping linear relationship at lower water potentials, and a horizontal relationship at high water potentials. That is, for dilute solutions (near 0 kPa), water uptake rates were similar despite the frogs being subjected to different water potentials, but at more negative water potentials there was a sloping linear relationship between water potential and water uptake. Thus, because it was clear that different mechanisms governed the points at different water potentials for these species, only the points along the slope were used in regression analyses to determine the seat patch water potentials (intercept of the *X*‐axis). In *P*. *cadaverina*, the *X*‐intercept was not different if the point for the highest water potential was used in the regression or not. We used an ANOVA followed by a Tukey HSD to test for differences of seat patch water potentials among the six species.

### 2.3 Osmotic potential of blood

Several months after the experiments described above were completed, the osmotic potential of the blood was measured for each frog at 100% and 90% of their standard (fully hydrated) body mass, with a one‐week interval between the two measurements. Although most species were allowed to dehydrate in air, *X*. *laevis* had to be dehydrated osmotically by placing them in a sucrose solution of −600 kPa because this species showed signs of distress in air (perhaps they cannot supply mucus to the skin as can more terrestrial anurans). Blood samples (approximately 25 μl) were collected from all frogs via the abdominal midline vein using a 23‐gauge hypodermic needle, and whole blood osmolality was measured immediately with a freezing point osmometer (Advanced Instruments 3MO).

Water potential is expressed in the SI units of kPa, but the osmometer output was in units of mOsm. Blood osmotic potentials were converted to units of kPa using the van't Hoff law (Salisbury & Ross, 1969).

The osmotic potentials of blood for each species at 100% and 90% hydration were compared using a paired t‐test. For each species, the water potential of the seat patch and the osmotic potential of blood at 90% hydration were also compared using a paired *t*‐test.

## 3 RESULTS

### 3.1 Water exchange with the environment

**FIGURE 1:**
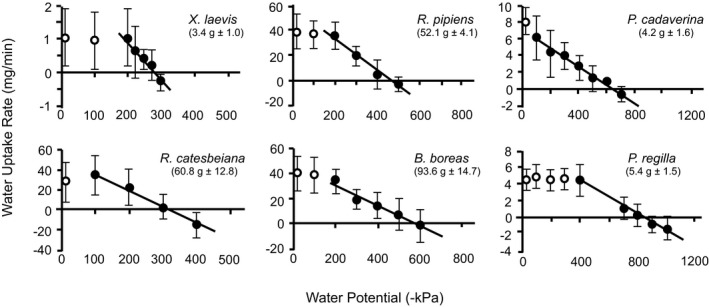
The rate of water exchange, corrected for cutaneous evaporative water loss, for six anuran species (mean ± SD) exposed to different environmental water potentials (sucrose solutions). The *x*‐intercept of the slope is taken to be the point at which no net water exchange occurs and is therefore where the water potential of the seat patch (or ventral site of water uptake) is equal to the environmental water potential. Data points are mean water uptake rates for all frogs at a given environmental water potential. The linear equations for the rate of water exchange between a frog and its environment were calculated using the filled circles, whereas the open circles represent points at higher water potentials at which water uptake rates were similar regardless of water potential. Mean and standard deviations of standard body masses are given under the names of species. Note that the whole‐body water uptake varies with body size, so the scales of Y‐axes differ among species

Evaporative water losses from agar model frogs in the experimental apparatus (Table 1) ranged from 0.42 mg min^−1^ in *X*. *laevis* to 4.7 mg min^−1^ in *R*. *catesbeiana*, representing, respectively, 0.88% to 0.17% of body mass lost per hour. These rates of evaporative water loss were added to species water uptake rates to account for water lost through cutaneous evaporation while in the experimental setup (Figure 1). Overall, the water uptake rates ranged from 43.01 ± 10.44 mg min^−1^ at 0 kPa for the terrestrial *R*. *pipiens* to −14.94 ± 13.85 mg min^−1^ at −400 kPa in *R*. *catesbeiana* (Figure 1).

**TABLE 1:** Mean (± SD) evaporative water loss (EWL in mg/min) of agar frog models (3% agar) made from six anuran species

Species	Mean EWL	*n* (mg/min)
*Xenopus laevis*	0.42 ± 0.02	4
*Rana catesbeiana*	4.7 ± 0.93	3
*Rana pipiens*	2.8 ± 0.42	4
*Bufo boreas*	2.5 ± 0.16	3
*Pseudacris cadaverina*	0.54 ± 0.15	4
*Pseudacris regilla*	0.53 ± 0.06	4

Note

These values of EWL were used to correct the values of water exchange shown in Figure 1. Mean body masses of the frogs used to make the models are given in Figure 1.

### 3.2 Water potential of seat patches and blood

**FIGURE 2:**
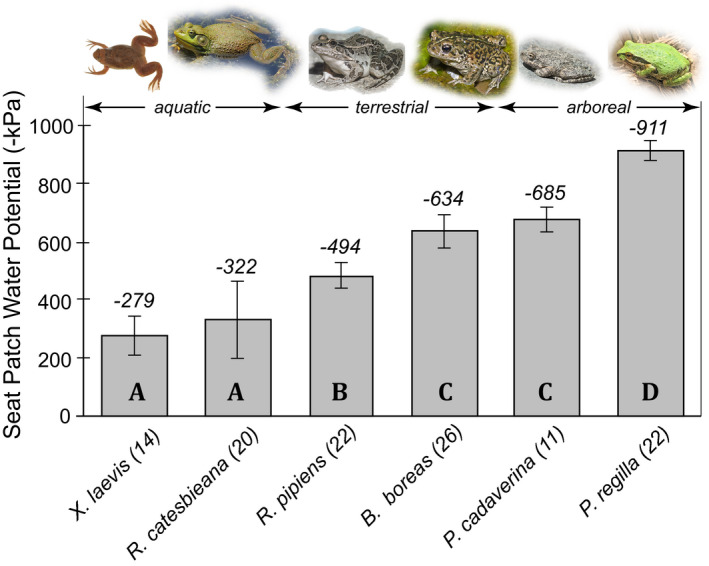
The water potential of the seat patches (or ventral sites of water uptake) (mean ± SD) of six anuran species dehydrated to 90% standard body mass. Sample sizes are given in parentheses. Species have been arranged in order of increasing terrestriality from left to right. Similar letters in the histogram bars indicate no significant difference between bars determined by analysis of variance with HSD post hoc tests at the 0.05 level. Pictures of frogs mostly came from thumbnails of photos available on AmphibiaWeb: Cal Photos. 2021. Regents of the University of California, Berkeley. Accessed June 7, 2021. Available online at: https://calphotos.berkeley.edu/. The photo of *Pseudacris regilla* was modified from a picture from Dr. Bobby Espinoza at CSUN

Seat patch water potentials were generally related to the ecological habit of the species (Figure 2). The seat patch water potential of the fully aquatic *X*. *laevis* was −279 ± 21 kPa and the semi‐aquatic *R*. *catesbeiana* was −322 ± 76 kPa. The terrestrial *R*. *pipiens* had a seat patch water potential of −494 ± 67 kPa, and the terrestrial *B*. *boreas* was −634 ± 144 kPa. The seat patch water potentials of the arboreal species were −685 ± 70 kPa for *P*. *cadaverina* and −911 ± 114 kPa for *P*. *regilla*.

**FIGURE 3:**
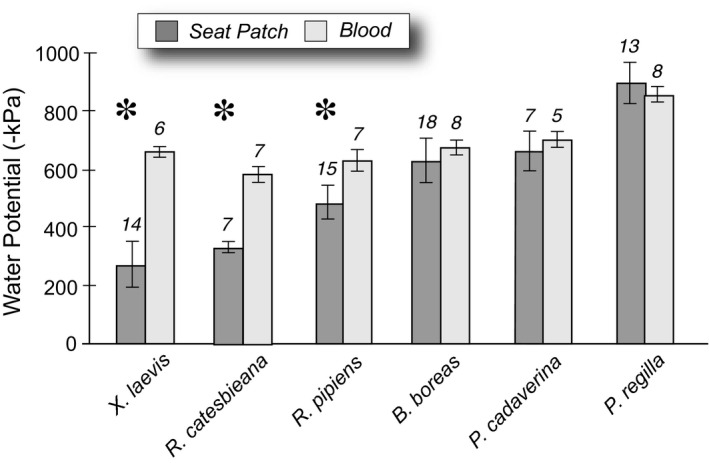
Comparisons between the water potentials of the seat patches (or ventral sites of water uptake) and the water potentials of their blood in six anuran species dehydrated to 90% of their fully hydrated mass. Data are means ± SD; * denotes statistical significance at *p* < .05 determined by analysis of variance and HSD posthoc tests

The osmotic potentials of blood (Table 2) were lower (more negative) at 90% hydration than at 100% hydration, indicating that they had a greater concentration of osmolytes. An ANOVA evaluating differences in water potentials of the seat patches and blood of all six species was highly significant (*F*_11, 103_ = 53.46, *p* < .0001) with a significant difference between water potential of seat patch and blood (*F*_5, 109_ = 95.36, *p* < .0001), and a significant interaction between species and source of water potential (viz., seat patch or blood); (*F*_5, 109_ = 17.89, *p* < .0001). A posthoc analysis (Tukey HSD) showed that the more aquatic species *X*. *laevis*, *R*. *catesbieana*, and *R*. *pipiens* had significant differences (Q = 3.34, *p* < .05) between their seat patch water potential and their blood water potential (Figure 3), with seat patch water potentials being higher (less concentrated) than blood water potentials. However, in the remaining terrestrial and arboreal species, the seat patch water potentials were not different from that of their blood.

**TABLE 2:** Osmotic potentials of blood (mean ± SD) in six anuran species while at 100% and 90% standard body mass (the mass of a fully hydrated frog with an empty bladder) as measured using a freezing point osmometer

Species	Osmotic Potential (‐kPa)	
	Blood (100%)	Blood (90%)
*Xenopus laevis*	581 ± 23 (6)	686 ± 29 (7)d
*Rana catesbeiana*	480 ± 32 (12)	580 ± 13 (6)d
*Rana pipiens*	593 ± 22 (7)	635 ± 31 (7)c
*Bufo boreas*	590 ± 7 (6)	685 ± 11 (8)b
*Pseudacris cadaverina*	635 ± 35 (4)	707 ± 13 (3)b
*Pseudacris regilla*	710 ± 44 (6)	842 ± E41 (9)a

Note

Sample sizes are given in parentheses. Water potentials at 90% hydration for all species were significantly higher (*t* = 1.995, *p* < .05) from those at 100% hydration. Letters designate osmotic potentials at 90% hydration that were statistically similar by Tukey HSD posthoc analysis (Q = 2.93, *p* < .05).

## 4 DISCUSSION

Seat patch water potentials were related to terrestriality (including arboreality) in the six frog species studied, with species that were more aquatic having higher (less negative) seat patch water potentials than those that were more terrestrial or arboreal. Similarly, differences between water potential of the seat patch and of the blood were related to terrestriality insofar as the aquatic species and the terrestrial *R*. *pipiens* had different osmotic potentials of the blood in comparison with their seat patch water potentials, but the terrestrial toad and arboreal species had osmotic potentials of the blood that were similar to those of their seat patches.

The basic physiology of water uptake through the ventral skin of amphibians has been studied extensively (reviewed by Jorgensen, 1997b) working under the assumption that blood osmotic water potential drives water exchange. Thus, the prevailing paradigm neither explains how aquatic frogs avoid taking up too much water, nor does it explain the disequilibrium between the seat patch water potential and blood water potential in species that live in or near water (Figure 3). Studies of water uptake in amphibians have emphasized the behavioral, physiological, and ecological mechanisms that allow “thirsty” terrestrial frogs to take up water rapidly (Jorgensen, 1997b; Tracy, 1976) with little emphasis on how frogs in aquatic environments avoid taking up too much water.

Our results suggest that species that are more aquatic appear to control their seat‐patch water potentials to be higher (less concentrated) than the osmotic potential of their blood, but species that are more terrestrial appear unable to do so. This ability to control the water potential of the seat patch is potentially beneficial to aquatic species, as it would allow them to limit the influx of water. Unrestricted water influx into frogs that spend considerable time exposed to water could create metabolic and electrolytic costs associated with ridding the body of excess water.

Terrestrial species face challenges to balancing their water budgets different from challenges faced by species that are primarily aquatic. For example, many terrestrial anurans absorb water from moist soils, and may be the primary source of water for some terrestrial species. The water potentials of soils depend upon soil composition and water content of the soil (Rose, 1966; Tracy, 1976). Thus, terrestrial frogs would benefit from having a lower (more concentrated) seat‐patch water potential because that would allow absorption of water from soils with a wider range of water potentials, including relatively dry soils.

Although circulatory changes are associated with water uptake through the seat patch (Viborg & Hillyard, 2005; Viborg et al., 2006), it seems unlikely that circulatory adaptations alone could account for the disequilibrium between the seat patch and blood water potentials that we observed in the more aquatic species. The mechanisms by which frogs could regulate their seat patch water potential may involve hormonal control of water channel proteins called aquaporins (Preston & Agre, 1991) that are located in the bladder, in the tissues of the ventral skin, and possibly around the capillaries that connect the seat patch with the rest of the body's circulatory system. A number of different types of aquaporins have been described from various amphibian tissues, and there are differences among species with respect to aquaporin types and expression (Suzuki et al., 2015). We suggest that the conductance of the walls of the capillaries that connect the seat patch with the rest of the body's circulatory system can change in response to hormonal signals acting on aquaporins. This change in conductance regulates water uptake into the body's circulatory system. Furthermore, this regulation can, under some conditions (see below), result in a disequilibrium between the water potentials of the seat patch and the blood.

**FIGURE 4:**
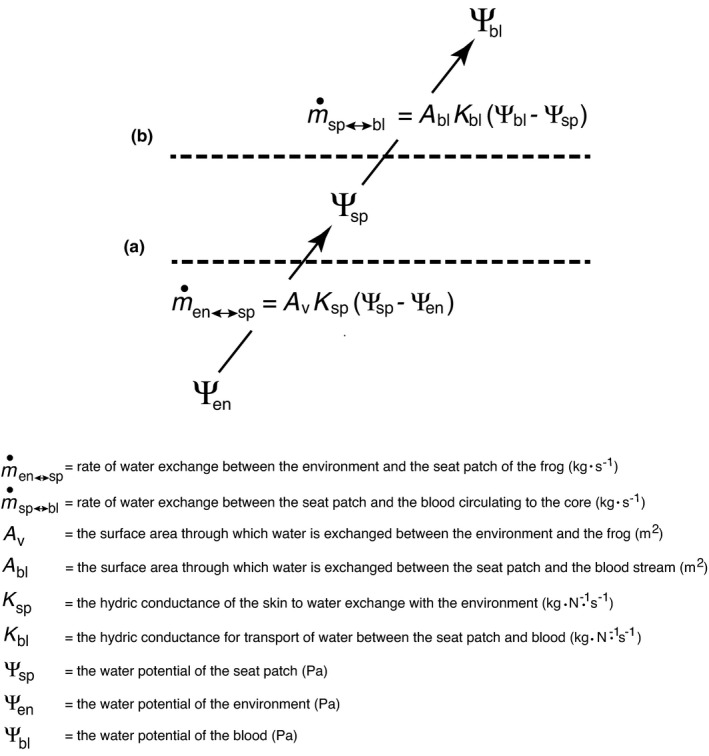
Model of water exchanges between the environment, seat patch (or ventral site of water uptake), and blood of a frog. The rate of water uptake from the environment through the skin surface (as described by Equation **A**) depends on the surface area of the seat patch (or ventral site of water uptake) that is exposed to water (*A*_v_), the hydric conductance of the skin (*K*_sp_), and the concentration gradient between the surrounding environment and the seat patch (or ventral site of water uptake) (Ѱ_sp_–Ѱ_en_). Similarly, water movement from the seat patch (or ventral site of water uptake) into the blood (as described by Equation **B**) depends on surface area, hydric conductance, and the concentration gradient between the seat patch and blood within the cutaneous blood vessels

The rates of water exchange between a frog and its environments can be modeled with the equations in Figure 4 (Tracy, 1976, 1982; Tracy & Rubink, 1978). If all properties, or system states, of Equation **A** (viz., *A*_v_, *K*_sp_, *Ψ*_sp_) are constant, then equation **A** describes a straight line where the slope of the line is (*A*_v_ * *K*_sp_), and the x‐intercept is where *Ψ*_sp =_ *Ψ*_en_. However, in experiments in which the environmental (sucrose) water potential was a variable, our results (in Figure 1) indicate that the lines, for most species, are not uniformly straight lines, but that they change slope relative to environmental water potentials. Thus, *K*_sp_ is a constant at more negative environmental water potentials (closed circles in Figure 1), but in environments with osmotic potentials closer to pure water there was a variable conductance of the frog (*K*_sp_) sufficient to offset the changing environmental (sucrose) water potential, leading to the observed leveling of the relationship between water uptake and environmental water potential (open circles in Figure 1). It seems likely that this pattern is due to changes in aquaporin activity that prevents uptake of excess water in dilute environments. This indicates that water potential of seat patches (likely due to hormonally controlled aquaporin activity) and hydric conductances of seat patches are variables that appear to act differently among different species, related to their ecological habits.

If water uptake rates are slowed in environments with higher water potentials (i.e., water potentials closer to that of pure water) by the actions of aquaporins located between the seat patch and the circulatory system (Equation **B** in Figure 4), this would provide a mechanism that would explain the data designated by open circles in Figure 1. Five of the six species (all except *P*. *cadaverina*) maintained a relatively similar water uptake rate while immersed in a range of high water potentials. Thus, if the flow of water from the seat patch to the circulatory system was restricted by the controlling action of aquaporins, the seat patch water potential (*Ψ*_sp_) would be high and similar to the environmental water potential, but the water potential of the blood would remain low (i.e., the blood would maintain its osmotic concentration) rather than becoming excessively dilute. This (possibly aquaporin‐mediated) regulation would be particularly beneficial for amphibians that spend a lot of time in water, and is consistent with our results (Figures 1, 2, and 3) that show that the more aquatic species have fluids in the seat patch that are significantly more dilute than blood. Such a disequilibrium cannot be explained simply by the water potential gradients between the seat patch and the blood, but it could result from an aquaporin‐mediated decrease in hydric conductance between the seat patch and blood (*K*_bl_). As mentioned above, there is evidence that the influx of water across the skin is independent of cutaneous blood flow (Viborg & Hillyard, 2005; Viborg et al., 2006). However, Burggren and Vitalis (2005) have raised the possibility that blood flow may play a role in regulating water influx by changing the “functional” surface area (i.e., A_bl_ in Equation **B** in Figure 4). Thus, it is possible that blood flow may play a role in regulating rates of water uptake in addition to changes in conductance (by way of aquaporins).

Word and Hillman (2005) provide evidence that water taken across the skin of cane toads (*Bufo marinus*) is taken directly into the capillaries rather than accumulating in lymphatic spaces. As we have shown in Figure 3, for the boreal toad, the water potentials of the blood and seat patch are not different. Thus, there is no barrier preventing water being taken into the capillaries, so we would not expect an accumulation of fluids in the lymph. Therefore, the result for cane toads is wholly consistent with our results for boreal toads and with the model in Figure 4. However, our model predicts that there would be an accumulation of dilute fluids in the ventral tissues in aquatic frogs such as *Xenopus* – a hypothesis that could be tested using the techniques of Word and Hillman (2005). An alternative hypothesis would be that the relatively impermeable skin of *Xenopus* could be explained by a lack of AVT‐stimulated aquaporins in this species. However, the lack of an AVT response does not explain what mechanism would prevent osmotic dilution of blood over time. More importantly, in the context of the results shown in Figure 1, a generalized low permeability of *Xenopus* skin might explain the low rate of water uptake, but it would not explain the less negative water potential (compared to the other species in our study) represented by the 0 uptake point on the *X*‐axis intercept. This less negative water potential suggests that there is an accumulation of dilute fluid in *Xenopus*. Thus, the aquaporin‐mediated model in Figure 4 is consistent with both the ability of terrestrial frogs to take up water from a relatively dry environment, and the ability of aquatic frogs to prevent an excessive water influx from a freshwater environment.

What currently is known about the types and roles of aquaporins in frog skin, bladders, and kidneys has been reviewed by Suzuki et al. (2007), Suzuki and Tanaka (2009), Ogushi, Akabane, et al. (2010), Ogushi, Tsuzuki, et al. (2010), and Suzuki et al. (2015). These reviews, based on data from relatively few species of frogs, present a mechanistic aquaporin model for the transport of water across frog epidermis that involves hormonal control of three or more different aquaporin types. Although the model describes the transport of water from the environment across the epithelium, it does not explain the control of water transport into the sub‐epidermal capillaries that link the seat patch fluids with the circulatory system of the body. Thus, the aquaporin model for cutaneous water uptake is incomplete. Much like the historical paradigm of water uptake that assumed that osmotic water potential of blood drives water exchange, the current aquaporin model explains how terrestrial frogs take up water, but it does not explain how aquatic species avoid taking up too much water. To address this fundamental question, we need to know more about aquaporin types from a wider range of species, and more about the mechanisms that control their levels of expression. Furthermore, to achieve a comprehensive understanding of frog water balance, the physiological and molecular studies of aquaporins should be designed within an ecological context by carefully selecting the species studied.

Evaluating the mechanisms by which the biophysical variables illustrated in Figure 4 change to produce an osmotic disequilibrium in different parts of a frog is beyond the scope of this report, but it represents an exciting and logical next step in investigating the physiology of water uptake in frogs. An integrated approach involving physiological, molecular, and ecological principles is needed to understand the evolutionary adaptations for water exchange between frogs and their environments.

## CONFLICT OF INTEREST

None declared.

## AUTHOR CONTRIBUTIONS

**Lee A. Lemenager:** Data curation (equal); Formal analysis (equal); Funding acquisition (supporting); Investigation (equal); Methodology (equal); Project administration (equal); Resources (supporting); Software (supporting); Supervision (equal); Validation (equal); Visualization (equal); Writing – original draft (equal); Writing – review & editing (equal). **Christopher R. Tracy:** Conceptualization (equal); Data curation (supporting); Formal analysis (equal); Funding acquisition (supporting); Investigation (equal); Methodology (equal); Project administration (supporting); Resources (equal); Software (equal); Supervision (supporting); Validation (equal); Visualization (equal); Writing – original draft (equal); Writing – review & editing (equal). **Keith Christian:** Conceptualization (equal); Data curation (supporting); Formal analysis (equal); Funding acquisition (equal); Investigation (equal); Methodology (equal); Project administration (supporting); Resources (equal); Software (equal); Supervision (supporting); Validation (equal); Visualization (equal); Writing – original draft (equal); Writing – review & editing (equal). **C. Richard Tracy:** Conceptualization; Data curation (equal); Formal analysis; Funding acquisition (lead); Investigation (equal); Methodology (equal); Project administration (lead); Resources (lead); Software (equal); Supervision (lead); Validation (equal); Visualization (equal); Writing – original draft (equal); Writing – review & editing (equal).

## Data Availability

All water potential data have been deposited in Dryad at: https://doi.org/10.5061/dryad.mpg4f4r0w.
